# Autonomic response and attachment style in disruptive mood dysregulation disorder

**DOI:** 10.4102/sajpsychiatry.v30i0.2293

**Published:** 2024-10-28

**Authors:** Michelle Leal, Marilyn Adan, Keri J. Heilman, Kate Cockcroft

**Affiliations:** 1 Department of Psychology, Faculty of Humanities, University of the Witwatersrand, Johannesburg, South Africa; 2 Department of Psychiatry, Faculty of Medicine, University of North Carolina, Chapel Hill, United States

**Keywords:** DMDD, emotion regulation, attachment, heart rate variability, vagal tone

## Abstract

**Background:**

Disruptive mood dysregulation disorder (DMDD) is characterised by severe emotion regulation difficulties, particularly anger and irritability, in children. Despite the impact of attachment on emotional and behavioural regulation, the link between attachment style and DMDD is under-researched.

**Aim:**

This study investigated whether attachment style and parasympathetic regulation differentiate the response profiles to frustrating conditions between children diagnosed with DMDD and controls.

**Setting:**

Participants were assessed at schools in Johannesburg, Gauteng, South Africa.

**Methods:**

Thirty participants were divided into two matched groups (*n*_DMDD_ = 15; *n*_Control_ = 15) and assessed. Respiratory sinus arrhythmia (RSA) and heart period were measured during an Affective Posner Task, inducing frustration. Attachment style was assessed using the Attachment Style Classification Questionnaire for children. Parents of children with DMDD completed a social interaction problems questionnaire.

**Results:**

The DMDD group showed a higher prevalence of avoidant attachment style (*p* = 0.013) compared to controls. Both groups displayed adaptive vagal withdrawal and recovery during the task (*p* = 0.005; *p* = 0.021). Controls had significantly higher heart period throughout the assessment (game 1: *p* = 0.006; game 2: *p* = 0.013; game 3: *p* = 0.007). In the DMDD group, lower vagal tone during frustration correlated with more social interaction problems (*p* = 0.049).

**Conclusion:**

The study demonstrates a potential link between attachment style and altered physiological state in children with DMDD.

**Contribution:**

The findings provide insight into possible atypical vagal regulation of the heart and avoidant attachment styles in DMDD, highlighting potential therapeutic and intervention targets.

## Introduction

Disruptive mood dysregulation disorder (DMDD) is a childhood mood disorder that is characterised by severe difficulty in emotion regulation, particularly that of anger and irritability.^1^ In these children, dysregulation leads to frequent temper outbursts and persistent angry or irritable moods, with low frustration tolerance.^2^ A DMDD diagnosis can be made between the ages of 6 and 18 although the onset is typically before the age of 10.^3,4^

The inclusion of this diagnostic category in the Diagnostic and Statistical Manual of Mental Disorders, Fifth Edition (DSM-5) was met with considerable criticism regarding reliability, stability, comorbidity and pathologising normal childhood behaviour,^3,4^ which may have impacted its utility locally and internationally. Irritability, a key feature of DDMD, is cited as one of the most common reasons for families to seek child mental health services.^5^ Persistent irritability is also associated with higher levels of social impairment, school suspensions and service use.^6^ Young adults with a history of DMDD are at increased risk of impaired functioning in adulthood, compared to typically developing peers and individuals with a history of other childhood psychiatric disorders. Long-term outcomes of DMDD include being diagnosed with one or more psychiatric disorders, adverse health effects, being impoverished, negative encounters with law enforcement and low educational achievement.^7,8^ While the global prevalence of DMDD is low, ranging from 0.8% to 3.3%,^6^ many of the identified risk factors for DMDD are pervasive features in the lives of South African children,^9^ which may put them at an increased risk of developing this psychiatric disorder.

Studies investigating early risk factors for DMDD are limited. Available findings suggest that family history, adverse childhood experiences (ACEs) and nutritional status are predisposing factors associated with the risk of developing disruptive behaviour.^3^ In terms of family history, children of parents with psychopathology are at increased risk of developing DMDD.^10,11^ A history of parental depression and substance abuse is associated with higher rates of DMDD diagnosis and greater symptom severity.^12,13,14^ Adverse childhood experiences associated with an elevated risk of DMDD include not living with one or both biological parents, low parental education, poverty, abuse, trauma, grief, recent family relocation and malnutrition.^3,15,16^ More than half of South African adults have experienced ACEs, such as emotional or physical abuse and violence, during childhood, with an estimated 40% having experienced emotional neglect before the age of 18.^17,18^ The high prevalence of ACEs in South Africa necessitates an understanding of the relationship between these experiences and the onset and maintenance of disruptive behaviour. It is also important for disruptive behaviour that is pathological and warrants a DDMD diagnosis from that which occurs in typically developing children of the same age.

Much attention has been given to the protective effect of secure social bonds, particularly the affective bond between caregiver and infant (i.e. ‘attachment’^19^), on the developmental trajectories of psychopathology in children.^20,21,22,23^ Attachment, as defined in classical attachment theory, delineates the enduring emotional connection between an infant and its primary caregiver, with varying attachment styles (i.e. ‘secure’ or ‘insecure’) identified through the child’s responses to caregiver’s presence or absence.^24,25^ These attachment patterns serve as mechanisms for emotional regulation and the development of broader emotion regulation skills. From an evolutionary standpoint, attachment systems evolved to ensure survival via the co-regulation of physiological states, activated in response to distress and deactivated when proximity, security and comfort from the primary caregiver are available.^26,27^ Feeling ‘safe’ or securely attached allows the individual to explore the physical, emotional and social environment. When positive social bonds are not present, either because bonds were not formed or broken, the attachment system remains fully or partially activated. Accumulated experiences of unmet attachment needs could result in chronic activation of the attachment system and physiological state and behavioural dysregulation, including autonomic regulation of cardiac rate and even physical and mental illness.^28,29,30,31,32^ It is proposed that children with DMDD would show insecure attachment because of their prevailing emotions and moods of anger and irritability.

Respiratory sinus arrhythmia (RSA) is commonly used to assess the parasympathetic regulation of the heart. This phenomenon, where heart rate accelerates during inhalation and decelerates during exhalation, is recognised as a specific, valid, non-invasive and easily accessible measure of the myelinated vagal influence on the sinoatrial node.^33^ Respiratory sinus arrhythmia is well established in the literature and is increasingly applied in psychophysiological research as an indicator of the parasympathetic nervous system or cardiac vagal tone.^34,35^ According to the polyvagal theory, vagal tone is closely associated with emotional regulation and prosocial behaviour, making RSA a valuable tool for assessing the constructs examined in this study.^36,37^

### Aim and objectives

This study aimed to describe attachment styles and autonomic response patterns (i.e., vagal tone) to frustration in children diagnosed with DMDD in comparison to typically developing controls without a history of mental health problems. The hypothesis was that children with DMDD would differ in attachment scores and physiological state regulation compared to typically developing matched controls when faced with a frustrating task. The final objective was to examine the relationship between RSA and DMDD symptom severity, as reported by primary caregivers. The hypothesis was that reduced RSA would be associated with more severe symptoms.

## Research methods and design

### Setting

Participants were assessed in a single session at their respective schools in Johannesburg, South Africa, between 08:00 and 10:00.

### Sample population

This quasi-experimental study compared children diagnosed with DMDD to healthy age, gender and grade-matched controls. English-proficient participants, between the ages of 7 and 13, were recruited via purposive sampling (Table 1). Attention-deficit/hyperactivity disorder was the most prevalent comorbid disorder in DMDD participants (*n* = 14). Other comorbid disorders included conduct disorder (*n* = 1), anxiety (*n* = 1) and unspecified mood disorder (*n* = 1). Exclusion criteria for this group were compromised neurology (e.g. epilepsy) or serious illness (e.g. meningitis) in the last 3 months. Control group exclusions included meeting any of the Diagnostic and Statistical Manual of Mental Disorders, Fifth Edition (DSM-5) criteria for DMDD or having immediate family members with a history of mental health conditions (e.g. depressive or anxiety disorders) to minimise potential genetic and environmental counfounds.^38^ The final sample comprised 30 children and adolescents, who were matched on age, gender and ethnicity (15 per group; 14 male; MAge_Control_ = 10.13, SDAge_Control_ = 1.85; MAge_DMDD_ = 10.13, SDAge_DMDD_ = 1.73). Sample size was determined using G*Power, aiming to detect medium to large effect sizes between the DMDD and control groups. Practical constraints limited the study to 30 participants, which was sufficient to detect medium effect sizes (*dz* ≥ 0.53).

**TABLE 1:** Demographic variables, by group (*N* = 15).

Variable	Category	Control	%	DMDD	%
Gender	Male	14	93	14	93
	Female	1	7	1	7
Ethnicity	African	5	33	5	33
	Caucasian	7	47	7	47
	Indian	1	7	1	7
	Mixed	2	13	2	13
Home language	African	4	27	5	33
	English	11	73	10	67
Medication	CNS stimulants (Methylphenidate)	-	-	11	73
	Atypical antipsychotics (Aripiprazole, Risperidone)	-	-	13	87
	Anticonvulsant (Lamotrigine)	-	-	2	13
	SSRIs (Escitalopram, Fluoxetine)	-	-	6	40
	Tricyclic antidepressants (Imipramine)	-	-	1	7
Medical aid	Yes	11	73	8	53
	No	4	27	7	47
Parent 1 Highest level of education	Further	5	33	11	73
	Higher	9	60	2	13
	Other	1	7	2	13
Parent 2 Highest level of education	Further	4	27	9	60
	Higher	9	60	-	-
	Other	1	7	2	13
	Missing	1	7	4	27

*Source:* Leal M. Disruptive Mood Dysregulation Disorder: a polyvagal perspective (Doctoral dissertation)

Home language African = isiXhosa, isiZulu, Shona, siSwati; Further Education = grades 10, 11 and 12; National Qualifications Framework (NQF) levels 2 (Certificate), 3 (Certificate) and 4 (Diploma). Higher Education = NQF levels 5 (Certificate, Higher Certificate and First Diploma), 6 (Bachelor’s degree, Professional first degree postgraduate, and first degree), 7 (Postgraduate Diploma, Honours Degree, and Master’s Degree), and 8 (PhD); DMDD, Disruptive Mood Dysregulation Disorder.

### Study design

**FIGURE 1:**
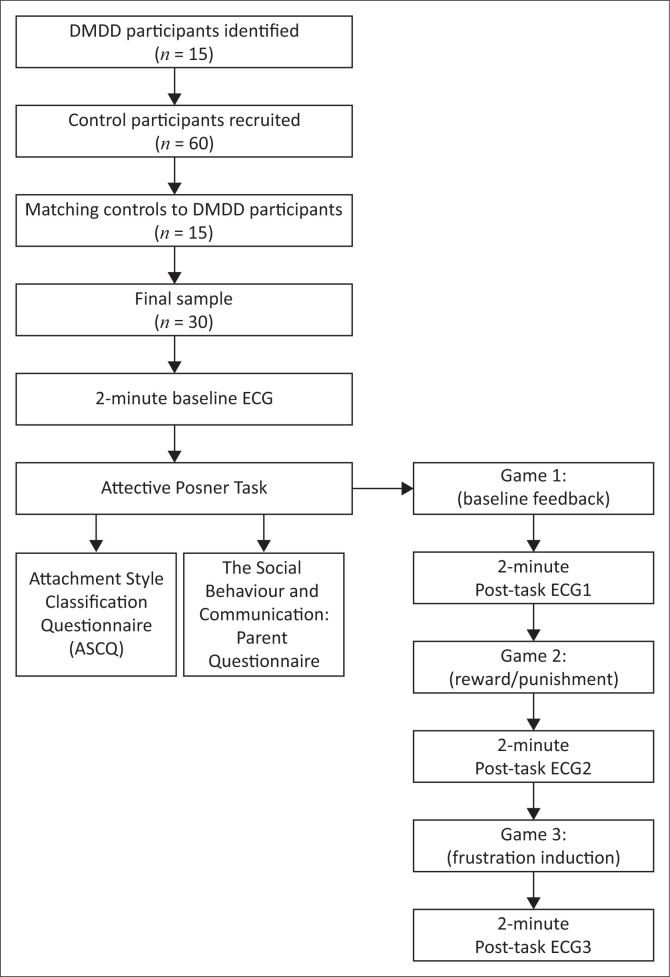
Flow chart of participant recruitment and study design for Disruptive Mood Dysregulation Disorder research.

The study’s main outcome was to establish whether attachment style and the parasympathetic component of the autonomic nervous system differentiate the response profiles between children diagnosed with DMDD and controls when confronted with a frustrating task. A baseline electrocardiogram (ECG) was recorded before the commencement of the experiment and again after each condition of the experimental task (Figure 1). Participants then completed a self-report measure of attachment style, and parents completed a questionnaire on their perceptions of their child’s social and behavioural difficulties.

The ECG was recorded at 1024 Hz using a wireless single-channel waveform recorder. The inter-beat interval (IBI) series was extracted from the ECG,^39^ examined visually undetected R-waves and artefacts and edited in CardioEdit.^40^ Editing was required for < 5% of each data file. CardioBatch^41^ was used to calculate RSA, which determines the contribution of the vagus nerve to overall heart period (HP). The detrended residual series underwent band-pass filtering to isolate HP pattern variance at the respiratory frequency for children (0.12–1.0 Hz).

A rigged version of the Affective Posner Task^42,43^ test elicited frustration in participants by manipulating feedback about response time. The task involved three games with various trials, each consisting of visual cues, targets and feedback. Game 1 served as a baseline with accurate feedback but no reward or punishment. Game 2 provided accurate feedback and rewarded or punished performance. Game 3 induced frustration by giving negative feedback regardless of performance. Frustration was induced only in the final game to increase arousal gradually and prevent carry-over.

The Attachment Style Classification Questionnaire (ASCQ)^44,45^ assessed participants’ attachment styles. It comprises 15 items, divided into three factors that correspond to Ainsworth’s^25^ attachment patterns. These categories include secure attachment (e.g. ‘I usually believe that others who are close to me will not leave me’), anxious or ambivalent attachment (e.g. ‘I’m sometimes afraid that no one really loves me’) and avoidant attachment (e.g. ‘I find it uncomfortable and get annoyed when someone tries to get too close to me’). Responses were scored on a 5-point Likert scale (1 = ‘All wrong’, 5 = ‘Very right’). Avoidant and anxious attachment scores were calculated by averaging their respective items.

The Social Behaviour and Communication: Parent Questionnaire, adapted from the Listening Project Parent Questionnaire,^46^ is an informal structured assessment tool. It evaluates parental observations of their child’s developmental and behavioural challenges across five areas: gestural and facial expression; language and social interaction; auditory processing; emotional regulation and reciprocity and behaviour. It encompasses areas such as hearing sensitivity, speech, spontaneity, emotional control and relatedness (Table 2). Parents reported any difficulties and changes in their child’s behaviour since starting medication, providing examples where possible. Scores ranged from -1 to 1, indicating improvement (-1), no or vague improvement (0) or deterioration (1) in different domains. The total count of problems was determined for each domain.

**TABLE 2:** Behavioural domains and explanations for the structured parent questionnaire.

Definitions	Description
Hearing sensitivity	Exaggerated negative responses (e.g. crying or placing hands over the ears) to common noises (e.g. vacuum cleaner, garbage disposal, baby crying, and air conditioning)
Spontaneous speech	Non-prompted use of words and sentences to communicate thoughts and ideas
Receptive speech	Ability to understand instructions and phrases
Spontaneity	Non-prompted behaviours initiated by the child
Behavioural organisation	Ability to occupy oneself (when left alone) in a productive and non-stereotypical way
Emotional control	Ability to calm quickly when upset, to respond to unexpected changes without getting upset, and to tolerate objections and contradictions of other people
Affection	Behaviours reflective of warm emotional state expressed by the child towards familiar people (e.g. hugging, kissing, and saying ‘I love you’ to the parent)
Listening	Ability to focus on human speech without visual or contextual cues, to understand spoken words, and to follow verbal requests
Eye contact	Making and maintaining eye contact during social interactions
Relatedness	Non-prompted social behaviours that reflect understanding of a joint partnership in interactions and sharing the same goals during social interactions (e.g. looking at a partner, showing toys, sharing an idea or a thought, and directing emotions to the partner)

*Source:* Reprinted from Porges SW, Bazhenova OV, Bal E, et al. Reducing auditory hypersensitivities in autistic spectrum disorder: Preliminary findings evaluating the listening project protocol. Front Pediatr. 2014;2:80. https://doi.org/10.3389/fped.2014.00080. Copyright © 2014 Porges, Bazhenova, Bal, Carlson, Sorokin, Heilman, Cook and Lewis

### Data analysis

Baseline demographic and clinical variables not used for matching were compared between groups using the paired *t*-test for years of exposure to English and McNemar’s test for paired categorical data for the remaining variables.

The effect of group on each of the attachment scores was determined using a repeated measures mixed model with attachment score as the dependent variable and group as independent variable. Group was treated as a repeated measure to capture the paired (matched case-control) nature of the data.

To determine whether the DMDD and matched control groups differed in their autonomic reactivity during the Affective Posner Task, a repeated measures mixed effects model was employed with the outcome (i.e. RSA or HP) as dependent variable and group, test (i.e. game 1, game 2 and game 3) the group-test interaction and age as independent variables.^47^ Group was treated as a repeated measure. Autonomic reactivity was assessed as the difference between baseline and RSA and HP measured during each game. In all analyses, non-normality of the model residuals was established by inspecting model diagnostics. Post hoc comparisons used the Tukey-Kramer adjustment for multiple comparisons.

The correlation between the number of social interaction problems and RSA for Game 3 of the Posner task was measured by Spearman’s correlation coefficient (as data were not normally distributed).

### Ethical considerations

Ethical clearance to conduct this study was obtained from the University of the Witwatersrand Human Research Ethics Committee (Medical) (No. M150526) and the Gauteng Department of Education (No. D2016/049, D2016/335A and D2017/319AA). Participants and their legal guardians gave written informed consent before participation, with appropriate opportunities for withdrawal without prejudice. With permission from the Ethics Committee, participants were not informed of the frustration-inducing aspect of the study, which involved some misleading information. All participants were debriefed immediately after the experiment. Anonymity and confidentiality of results were assured.

## Results

### Demographics

There were no significant group differences in the number of years of exposure to English (F(1, 12.9) = 3.48, *p* = 0.085), home language (χ^2^(1) = 0.33, *p* = 0.56), medical aid (χ^2^(1) = 1.29, *p* = 0.26) or parental level of education ((${\text{χ}}_{1}^{2}$(3) = 4.83, *p* = 0.18; ${\text{χ}}_{1}^{2}$(1) = 2.67, *p* = 0.10); Table 1).

### Attachment style

Disruptive mood dysregulation disorder participants scored significantly higher on avoidant attachment than controls (DMDD: Least squares mean (LSM) = 3.0, 95% CI 2.5–3.5; Control: LSM = 2.0; 95% CI 1.5–2.5; *d* = 0.98). There were no significant group differences in anxious attachment scores (DMDD: LSM = 2.9, 95% CI 2.5–3.0; Control: LSM = 2.5; 95% CI 2.1–2.9).

### Heart rate variability measures

Table 3 summarises the descriptive statistics for each group’s RSA and HP measurements before, during and following administration of the Affective Posner Task.

**TABLE 3:** Descriptive statistics for the affective Posner task physiological measurements (in milliseconds), by group (*N* = 15).

Condition	Control				DMDD			
	M	s.d.	Mdn	IQR	M	s.d.	Mdn	IQR
**RSA ln (msec)^2^**								
Pre-task Baseline	8.05	0.89	8.01	7.09–9.16	7.39	1.26	7.54	6.59–8.26
Practice	8.10	1.16	8.02	7.36–9.13	7.23	1.18	7.38	6.77 – 8.02
Game 1	7.76	1.06	7.62	6.91–8.74	7.05	1.10	7.15	6.85–7.61
Game 1 Recovery	7.93	1.01	7.77	7.03–8.66	7.22	1.03	7.21	6.67–8.16
Game 2	7.68	1.05	7.58	6.96–8.67	7.18	0.95	7.31	7.11–7.70
Game 2 Recovery	8.01	0.93	7.99	7.28–8.73	7.66	1.59	7.17	6.64–8.80
Game 3	7.81	0.98	7.81	7.24–8.55	7.30	1.00	7.45	6.70–7.90
Game 3 Recovery	8.00	0.86	8.05	7.06–8.71	7.45	1.18	7.34	6.72–8.88
**HP (msec)**								
Pre-task Baseline	759.69	82.64	768.29	687.52–807.40	671.71	86.74	653.80	618.78–676.62
Practice	761.38	74.60	763.58	714.09–806.43	681.76	91.86	662.38	617.85–714.91
Game 1	738.51	76.14	747.08	692.93–779.32	666.86	83.49	644.29	621.88–717.14
Game 1 Recovery	748.91	81.36	733.86	701.13–786.07	671.81	79.33	634.84	625.09–703.62
Game 2	732.17	71.63	736.75	691.63–797.81	677.63	96.08	644.48	621.88–719.12
Game 2 Recovery	746.72	77.91	741.54	693.93–804.46	684.41	93.01	640.17	624.60–782.20
Game 3	739.29	75.16	733.70	708.22–780.79	679.87	83.73	647.10	633.14–722.04
Game 3 Recovery	742.18	71.88	746.03	698.25–802.25	668.44	70.26	646.92	634.76–688.87

*Source:* Leal M. Disruptive Mood Dysregulation Disorder: a polyvagal perspective (Doctoral dissertation)

M, Mean; s.d., Standard deviation; Mdn, Median; IQR, Inter-quartile range; RSA, Respiratory sinus arrythmia; HP, Heart period; DMDD, Disruptive Mood Dysregulation Disorder.

Controlling for baseline and age,^48^ there was a significant game effect for RSA in both groups, with a reduction in RSA from baseline to game 1 (F[1,26] = 9.20, *p* = 0.005; *d* = 0.40) and baseline to game 2 (F[1,26] = 6.07, *p* = 0.021; *d* = 0.42). The reductions in RSA from baseline to game 3 did not reach significance (Table 4). No significant group differences in RSA emerged from baseline to game 1, game 2 or game 3. Group-game interaction effects on RSA from baseline-to-game were also non-significant. No significant group, game or group-game effects emerged for RSA for game-to-recovery conditions.

**TABLE 4:** Inferential statistics for the affective Posner task.

Condition	Effect	*F*	*df*	*p*
RSA Baseline vs Game 1				
	Group	2.14	14	0.167
	Game	9.20	26	0.005*
	Group x Game	0.14	26	0.709
RSA Baseline vs Game 2				
	Group	1.83	14	0.013
	Game	6.07	26	0.200
	Group x Game	0.06	26	0.051
RSA Baseline vs Game 3				
	Group	1.57	14	0.231
	Game	2.00	26	0.169
	Group x Game	0.21	26	0.653
HP Baseline vs Game 1				
	Group	10.27	14	0.006*
	Game	4.12	28	0.052
	Group x Game	1.62	28	0.021
HP Baseline vs Game 2				
	Group	8.11	14	0.013*
	Game	1.74	28	0.197
	Group x Game	4.18	28	0.051
HP Baseline vs Game 3				
	Group	9.86	14	0.007*
	Game	0.53	28	0.471
	Group x Game	2.9	28	0.100
RSA Game 1 vs Game 1 Recovery				
	Group	2.87	14	0.112
	Game	1.62	26	0.214
	Group x Game	0	26	0.956
RSA Game 2 vs Game 2 Recovery				
	Group	2.40	14	0.144
	Game	3.42	26	0.076
	Group x Game	0.51	26	0.483
RSA Game 3 vs Game 3 Recovery				
	Group	1.28	14	0.276
	Game	3.16	26	0.087
	Group x Game	0.02	26	0.890
HP Game 1 vs Game 1 Recovery				
	Group	9.48	14	0.008*
	Game	1.65	28	0.209
	Group x Game	0.21	28	0.651
HP Game 2 vs Game 2 Recovery				
	Group	6.95	14	0.019*
	Game	1.01	29	0.326
	Group x Game	0.13	29	0.717
HP Game 3 vs Game 2 Recovery 3				
	Group	8.49	14	0.012*
	Game	0.64	28	0.430
	Group x Game	1.81	28	0.190

RSA, Respiratory sinus arrhythmia; HP, Heart period.

* , Statistically significant *p* < 0.005.

A significant group effect for HP was evident throughout all game conditions, with the control group consistently having a higher HP than the DMDD group. From baseline to game 1, the estimated LS-mean HP was 80 msec higher in controls compared to DMDD participants (F[1,14] = 10.27, *p* = 0.006; *d* = 1.24). Similar patterns emerged from baseline to game 2 (LSM = 71 msec higher; F[1,14] = 8.11, *p* = 0.013; *d* = 1.04) and from baseline to game 3 (LSM = 74 msec higher; *F*[1,14] = 9.86, *p* = 0.007; *d* = 1.16). The group effect on HP was also apparent during game-to-recovery conditions, with the control group exhibiting higher HP than the DMDD group (game to recovery 1: LSM = 74 msec higher; F[1,14] = 9.48, *p* = 0.008; *d* = 1.18; game to recovery 2: LSM = 58 msec higher; F[1,15] = 6.95, *p* = 0.019; *d* = 0.89; game to recovery 3: LSM = 67 msec higher; F[1,14] = 8.49, *p* = 0.012; *d* = 1.14) There were no significant game or group-game interaction effects on HP from baseline-to-game or game-to-recovery conditions (Table 4).

### The social behaviour and communication: Parent questionnaire

**FIGURE 2:**
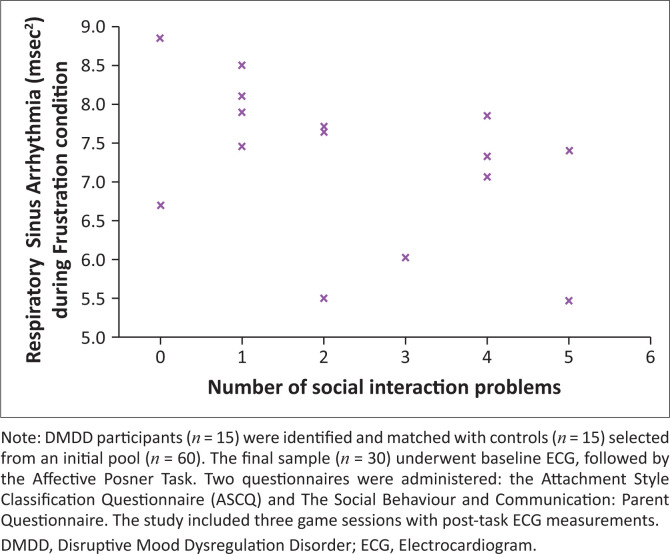
Correlation between Respiratory sinus arrhythmia during frustration condition and number of social interaction problems in Disruptive mood dysregulation disorder group.

Because of age-appropriate social skills in the control group, the analysis was limited to the DMDD group. A significant negative correlation was found between vagal tone (RSA) during frustration and social interaction issues (rho(28) = 0.52, *p* = 0.049; Figure 2). Children with lower vagal tone during the frustration condition had the highest score for interaction problems on the Social Behaviour and Communication: Parent Questionnaire.

## Discussion

This study explored attachment style and autonomic response profiles in a sample of South African children diagnosed with DMDD, compared to that of matched controls. Despite the identified risk factors for the development of DMDD,^3,10,11,12,13,14,15^ many of which could negatively affect attachment security, the relationship between attachment style and DMDD remains under-researched. One study found that children carrying a G allele (i.e. GG + AG genotypes) were more likely to have insecure attachment patterns and scored higher on subscales of Withdrawal and Conduct Problems than their homogenous AA-carrying counterparts.^49^ Another study examined the link between psychological symptoms, attachment style and global DNA myelination in healthy and DMDD mother-child pairs but found no significant effect for attachment style.^50^ In the current sample, avoidant attachment style was more prevalent in the DMDD group than in the control group. This finding aligns with literature that implicates the role of environmental input in emotion dysregulation and conduct problems^37^ and adds to the body of literature on attachment style in DMDD. It also suggests that interventions aimed at improving the caregiver-child bond could be helpful in terms of symptom management and improved emotion regulation.

While we expected that the DMDD group would exhibit different autonomic response patterns compared to typically developing matched controls, when faced with a frustrating task, this hypothesis was not supported by the results. In line with previous research, both groups exhibited adaptive reactivity^51,52,53^ to the first two tasks. Consistent with expectations, in both groups, RSA decreased during game 1 (control condition) and game 2 (affective feedback) and increased during the recovery phases. Reactivity from baseline to game 3 was not significant for either group. This finding may reflect a shift in focus from the regulation of internal processes to executive processes for problem-solving or enhanced attention.^54,55^ It may also indicate that the task was insufficiently frustrating or that habituation to the task occurred in the final game.^56^ Randomised delivery of the protocol should be implemented in future studies to minimise the effect of habituation.

Controls showed significantly higher heart period than DMDD participants across all assessment conditions, including baseline. This suggests heightened autonomic activity in DMDD participants, which has been reported in children with ADHD (comorbid in 14 of the DMDD participants) treated with stimulants.^57^ An increased heart rate (i.e. the inverse of heart period) may also indicate that the children in this group found the situation or the task more stressful than their peers, resulting in a ‘fight or flight’ state. The significant increase in autonomic activity in DMDD could be the focus of future research, particularly with the aim of tailored interventions for improving or regulating parasympathetic activity.

In both groups, changes in RSA were associated with inverse changes in HP, which indicates adaptive vagal regulation.^28,58^ In the DMDD group, children with lower vagal tone during the Affective Posner Task’s frustration condition were reported by parents to have more social interaction issues (Figure 2). This supports research that implicates vagal tone in social outcomes.^37^ The absence of attenuated vagal withdrawal during the frustration task suggests that children with DMDD have different autonomic responses when faced with frustration in social settings as opposed to frustration during a cognitive task. Further research is needed to determine whether autonomic response profiles of children with DMDD vary based on task type (i.e. cognitive versus social-based frustration tasks), which could also increase the ecological validity of the findings.

### Limitations

The results are limited by a few issues. Although not unusual for studies involving clinical populations,^11,59^ findings cannot be generalised because of the small sample size. Girls were underrepresented in the sample, which further limits the external validity of the findings. The study was conducted in Johannesburg, South Africa, and findings cannot be extrapolated to the wider South African context. Anthropometric factors (e.g., body weight, height, and waist-hip ratio) that affect RSA were not included because of the sample’s vulnerability and the settings where the data were collected (schools). Participants were also not medication naïve. Additionally, the lack of randomisation and blinding may have introduced selection and observer biases. Future studies should investigate the relationship between RSA and these variables in a similar sample.

## Conclusion

The study was the first South African study on DMDD. The study highlights the importance of understanding the link between biobehavioural regulation and clinical presentation in children who meet the criteria for DMDD, particularly in a socioeconomic environment where many are exposed to the risk factors of the disorder. The findings suggest that children who meet the criteria of DMDD are more likely to have avoidant attachment styles, supporting research on the moderating effect of secure attachment patterns on emotion regulation. They also exhibited higher sympathetic activation throughout the assessment. Furthermore, compared to typically developing children, those who meet the criteria for DMDD showed similar autonomic reactivity during the frustration condition. However, in this group, lower RSA was associated with more social interaction problems as reported by the parents. This is valuable knowledge for psychiatrists which can assist in providing personalised, effective and holistic care that addresses the child’s emotional, social and relational well-being.
